# Supplementary figures and images for: Identification of potential biomarkers of leprosy: A study based on GEO datasets
Source: PLoS One. 2024 May 13;19(5):e0302753. doi: 10.1371/journal.pone.0302753 (PMC11090354; doi:10.1371/journal.pone.0302753)

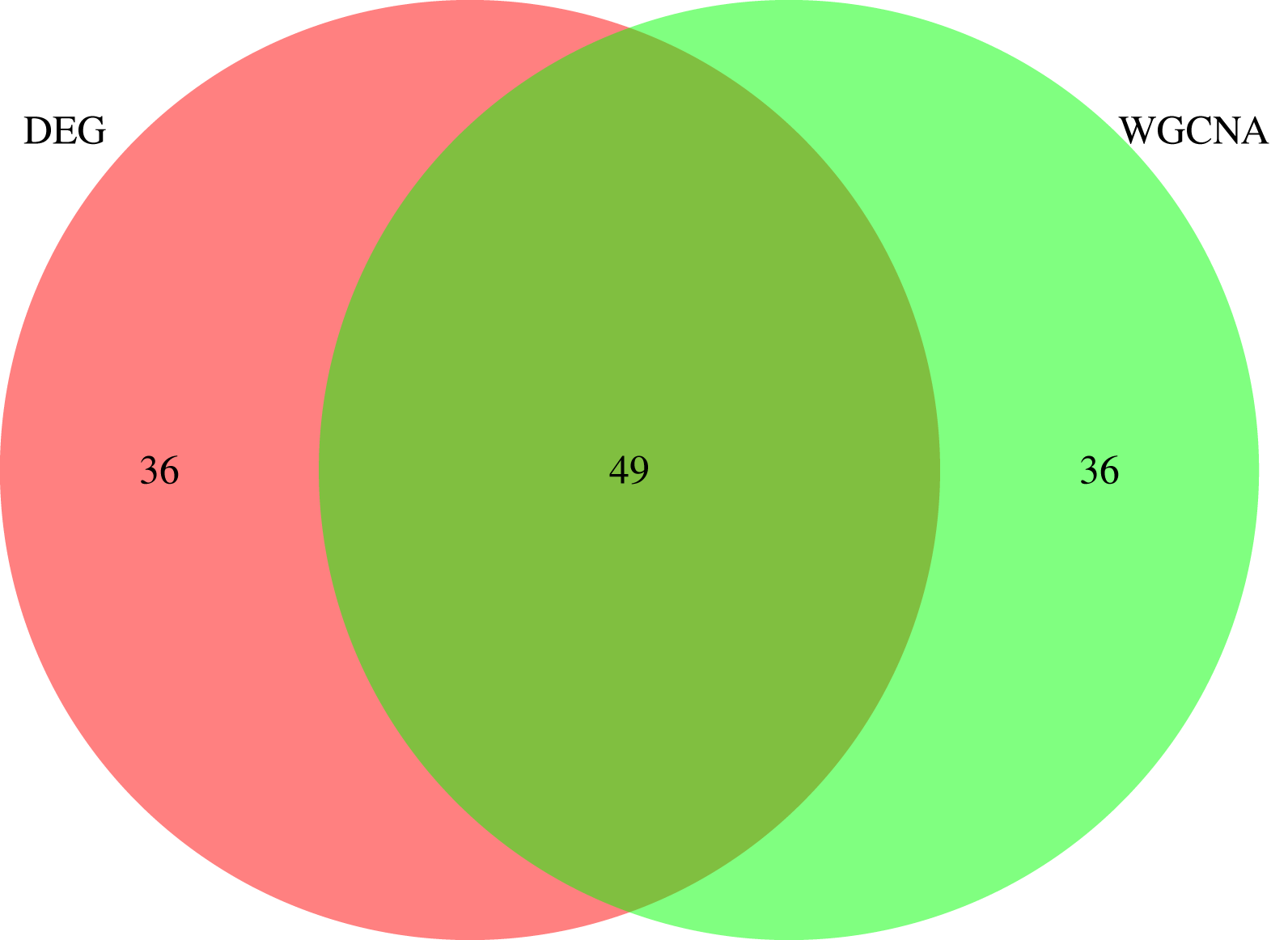

Supplement: S1 Fig — (TIF) [file pone.0302753.s007.tif]

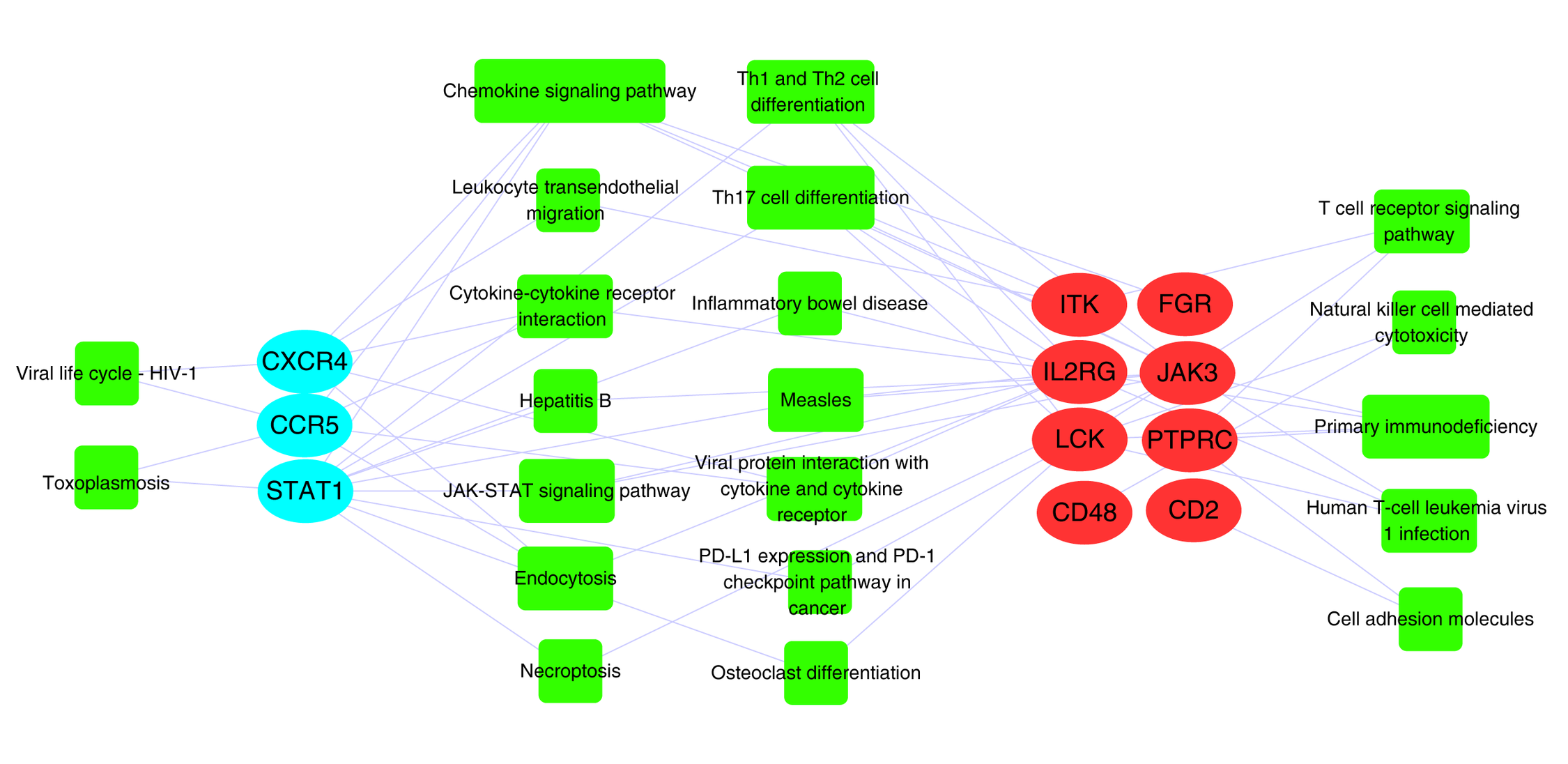

Supplement: S2 Fig — Oval box represents gene and square box represents pathway. Red color represents MCODE1 genes and blue represents MCODE2 genes. The wider the pathway frame, the more genes the pathway contains. (TIF) [file pone.0302753.s008.tif]

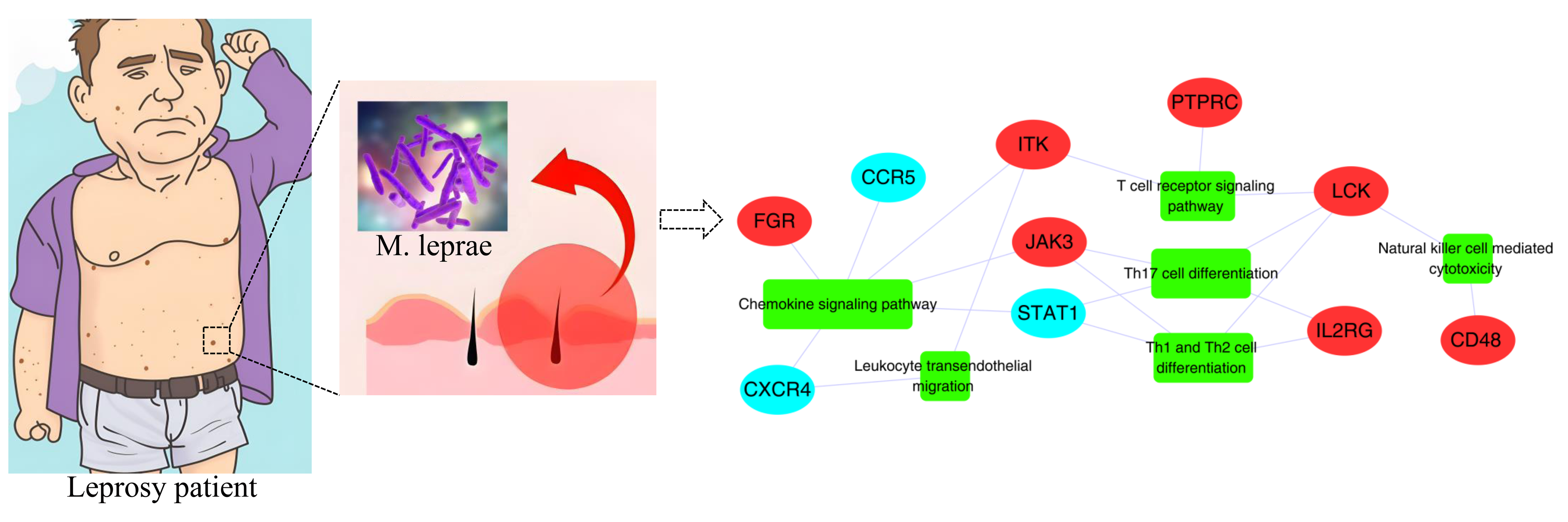

Supplement: S1 Graphical abstract — (TIF) [file pone.0302753.s009.tif]
