# Supplementary material for: Identification of potential biomarkers of leprosy: A study based on GEO datasets
Source: PLoS One. 2024 May 13;19(5):e0302753. doi: 10.1371/journal.pone.0302753 (PMC11090354; doi:10.1371/journal.pone.0302753)
Supplement: Attachment — Submitted filename: Bibhu_Review_ 25.10.2023.docx [file pone.0302753.s011.docx]

**Identification of crucial genes and functional network features of leprosy based on GEO expression profiles**

Here the authors have collected the bioinformatics analysis with leprosy and normal samples using GEO database. They have screened 85 hub genes using WGCNA. They performed DEG analysis and screened 82 up-regulated and 3 down-regulated genes. Finally, they have identified 10 genes (ITK, CD48, IL2RG, CCR5, FGR, JAK3, STAT1, LCK, PTPRC, CXCR4). They have suggested these 10 genes can be used as leprosy Biomarker because they were active in 6 immune system pathways, including Chemokine signaling pathway, Th1 and Th2 cell differentiation, Th17 cell differentiation, T cell receptor signalling pathway, Natural killer cell mediated cytotoxicity and Leukocyte trans endothelial migration. The research is exciting and valuable enough to be published in PLoS One. Before being accepted for publication, the piece must, however, be appropriately revised in accordance with the remarks offered below.

**Comments:**

1. The title of the manuscript should be modified.
2. If possible graphical abstract should be provided. According to journal format if it is not possible in the main text, it can be provided in the supplementary file for better understanding of the readers (optional).
3. The Abstract portion should be modified.
4. **Line Numbers should be added throughout manuscript.**
5. The introduction portion should be rewritten.
6. The author should give a strong hypothesis why this study in details.
7. **Introduction section:**

**(3^rd^ Paragraph) Line no -1:** detecte should be corrected.

8. **Materials and method section:**

**Here the authors have simply written they have taken the data and have used the different softwares for the data analysis but the detailed analysis regarding how it is doing that is completely lacking. This should be addressed properly.**

9. **Results section:**

All figures need a better resolution. The author should also mention the image source and the software used to make the images.

10. **Conclusion section:**

**The conclusion portion should be rewritten and the author must add a paragraph on future prospects**

11. After the conclusion section there should be some small sections like: Supporting information, Acknowledgments, Author Contributions etc
12. Correct the PubMed ID of Reference No. 40.

**Recommendation:** Major Revision
