# Supplementary material for: Identification of potential biomarkers of leprosy: A study based on GEO datasets
Source: PLoS One. 2024 May 13;19(5):e0302753. doi: 10.1371/journal.pone.0302753 (PMC11090354; doi:10.1371/journal.pone.0302753)
Supplement: Attachment — Submitted filename: Response to Reviewers.docx [file pone.0302753.s012.docx]

Dear Editors and Reviewers:

Thank you for your letter and for the reviewers’ comments concerning our manuscript entitled "Identification of crucial genes and functional network features of leprosy based on GEO expression profiles" (Manuscript ID: PONE-D-23-26444). We feel great thanks for your professional review work on our article. As you are concerned, there are several problems that need to be addressed. According to your nice suggestions, we have made extensive corrections to our previous draft, the detailed corrections are listed below.

**Response to Reviewer #1:**

Comments:

*For reproducibility's sake, I think, it'd be good if the authors make their R codes used for all analysis publicly available.*

Response to comment: We thank the reviewer for the kind comments. We will be happy to share the data with readers, and this effort can improve the reproducibility and citation of the article. As the software and its parameters used in the analysis have already been provided in the article, the data result can be easily reproduced. Moreover, we have provided the main codes used in the article as file “Main_workflow.R”. Additionally, the reader can contact the author to access more details.

**Response to Reviewer #2:**

Major comments:

1. *Please explain why the cutoffs and threshold limits (e.g. cutoffs for GS and MM, threshold for WGCNA analysis) were selected, including if similar studies implemented the same cutoffs.*

Response to comment: We thank the reviewer for the kind comments. We selected the cutoffs and threshold limits for their statistical significance. And our selections are evidence based. For example, we conducted WGCNA analysis and screened hub genes with the threshold at GS > 0.7 and MM > 0.8, because we can use gene significance (GS) to measure the correlation between genes and use modules and module membership (MM) to measure the correlation between genes and clinical traits. Weiwei Liang used GS > 0.2 and MM > 0.8 for hub genes(*Liang, W., Sun, F., Zhao, Y., et all. Identification of Susceptibility Modules and Genes for Cardiovascular Disease in Diabetic Patients Using WGCNA Analysis. Journal of diabetes research, 2020, 4178639.* [*https://doi.org/10.1155/2020/4178639*](https://doi.org/10.1155/2020/4178639)). We applied a stricter constraint to discover more meaningful genes.

2. *Please provide more explanation as to how the statistics were performed beyond the R package(s) utilized. Notably, the supplementary datasets contain statistical values that should be transferred to some capacity into the main text.*

Response to comment: Thank you for your suggestion, according to your suggestion, more explanation of how the statistics were performed beyond the R package(s) utilized has been added to the main text. The main analysis parameters of the software used in the analysis have already been provided in the article. (line 88, 101-103)

3. *Verbiage that was used to describe findings should be revised (e.g. revising “we got a gene-pathway network” to “we identified…”; “10 genes were finally got”, etc.)*

Response to comment: Thank you for your careful evaluation of this manuscript. We revised the title of the manuscript. (line 15, 197, 238)

Minor comments:

1. Please provide more explanation and description of the methods, including how/why each database was utilized.

Response to comment: We revised the manuscript according to the reviewer’s suggestion. In the portion of **Microarray Data from GEO data repository** in **Materials and methods**, why each database was utilized was described: the database was included in the study when it met the four criteria. In the portion of **Preprocessing of raw data** in **Materials and methods**, how each database was utilized was described: the selected five gene expression profiles were merged into one file, and then log and baseline transformations were done. More detailed description of other methods has been added to the main text. (line 88, 101-103)

**Reviewer #3: Comments:**
1. *The title of the manuscript should be modified.*

Response to comment: Thank you for your careful evaluation of this manuscript. We revised the title of the manuscript. (line 1-4)

2. *If possible graphical abstract should be provided. According to journal format if it is not possible in the main text, it can be provided in the supplementary file for better understanding of the readers (optional)*.

Response to comment: We adopt your suggestion, we have designed a graphical abstract as supplementary file.
3. *The Abstract portion should be modified.*

Response to comment: We have modified the Abstract part according to the Reviewer’s suggestion. (line 15-23)
4. *Line Numbers should be added throughout manuscript.*

Response to comment: We have added the Line Numbers according to the Reviewer’s suggestion.
5. *The introduction portion should be rewritten.*

Response to comment: Thank you for your suggestion, according to your suggestion, we revised the **Introduction** portion. (line 28-40, 57-62)
6. *The author should give a strong hypothesis why this study in details.*

Response to comment: Thank you for your suggestion, we have added some content to explain the aim of our work in the **Introduction** portion. (line 59-62)

7. *Introduction section: (3rd Paragraph) Line no -1: detecte should be corrected.*

Response to comment: It has been revised. (line 63)
8. *Materials and method section:
Here the authors have simply written they have taken the data and have used the different softwares for the data analysis but the detailed analysis regarding how it is doing that is completely lacking. This should be addressed properly.*
Response to comment: We totally agree with your suggestion. As the software and its parameters used in the analysis have already been provided in the article, the data result can be easily reproduced. Moreover, we have provided the main codes used in the article as file “Main_workflow.R”. Additionally, readers can contact the author to fetch the codes.

9. *Results section:
All figures need a better resolution. The author should also mention the image source and the software used to make the images.*
Response to comment: The high resolution images have been uploaded in Figures folder. The description of drawing software has been added in the main text. (line 88, 101-103)
10. *Conclusion section:
The conclusion portion should be rewritten and the author must add a paragraph on future prospects*

Response to comment: According to your suggestion, the conclusion portion has been rewritten and a paragraph on future prospects also has been added. (line 257-270)
11. *After the conclusion section there should be some small sections like: Supporting information, Acknowledgments, Author Contributions etc*

Response to comment: Sections (**Supporting information**, **Acknowledgments**, **Author Contributions**) have been added to the main text. (line 273-303)

12. *Correct the PubMed ID of Reference No. 40.*

Response to comment: PubMed ID of Reference No. 40 has been corrected. (line 400)

The reviewer specifically raised some concerns regarding the novelty and significance of our work. Based on the reviewers’ comments, we have made extensive alterations to the structure, format, presentation, and analysis of our findings. In response, we have addressed all of the reviewer’s technical concerns with a substantial amount of new data and graphic, supported by additional detailed explanations (the majority of the figures in both the text and supporting information have been revised as requested). If there are any other modifications we could make, we would like very much to modify them and we really appreciate your help. We hope that our manuscript could be considered for publication in Plos One. Thank you very much for your help.

Yours sincerely,

Yi Zheng

13 Jan, 2024
