# Supplementary material for: Identification of potential biomarkers of leprosy: A study based on GEO datasets
Source: PLoS One. 2024 May 13;19(5):e0302753. doi: 10.1371/journal.pone.0302753 (PMC11090354; doi:10.1371/journal.pone.0302753)
Supplement: Attachment — Submitted filename: Response to Reviewers.docx [file pone.0302753.s013.docx]

Dear Editors and Reviewers:

Thank you for your letter and for the reviewers’ comments concerning our manuscript entitled " Identification of Potential Biomarkers of Leprosy: A Study Based on GEO Datasets" (Manuscript ID: PONE-D-23-26444R1). We feel great thanks for your professional review work on our article. As you are concerned, there are several problems that need to be addressed. According to your nice suggestions, we have made extensive corrections to our previous draft, the detailed corrections are listed below.

Comments:

1. *Article is now in shape however needs further efforts to clinical relevance of study in discussion section. Comparative data or evaluation with another studies may give better insight.*

Response to comment: Thank you for your suggestion. We have added one paragraph to introduce relevant clinical research progress in discussion section. (line 251-258)

2. *Additionally, future prospect with better scope, affected region, populations, policy and technology relevance will connect it for better healthcare management.*

Response to comment: We adopt your suggestion, we have briefly introduced the current prevalence of leprosy. (line 268-270)

The reviewer specifically raised some concerns regarding the novelty and significance of our work. Based on the reviewers’ comments, we have made extensive alterations to the structure, format, presentation, and analysis of our findings. In response, we have addressed all of the reviewer’s technical concerns. If there are any other modifications we could make, we would like very much to modify them and we really appreciate your help. We hope that our manuscript could be considered for publication in Plos One. Thank you very much for your help.

Yours sincerely,

Yi Zheng

14 Mar, 2024
